# Effects of interdisciplinary pain rehabilitation programs on neuropathic and non-neuropathic chronic pain conditions – a registry-based cohort study from Swedish Quality Registry for Pain Rehabilitation (SQRP)

**DOI:** 10.1186/s12891-023-06462-2

**Published:** 2023-05-06

**Authors:** Nazdar Ghafouri, Emmanuel Bäckryd, Elena Dragioti, Marcelo Rivano Fischer, Åsa Ringqvist, Björn Gerdle

**Affiliations:** 1 Pain and Rehabilitation Centre, and Department of Health, Medicine and Caring Sciences, Linköping University, 58185 Linköping, Sweden; 2 Department of Neurosurgery and Pain Rehabilitation, Skåne University Hospital, 221 85 Lund, Sweden; 3 Department of Clinical Sciences, Faculty of Medicine, Lund University, Lund, Sweden

**Keywords:** Chronic pain, Interdisciplinary, Multimodal, Neuropathic pain, Outcome, Rehabilitation

## Abstract

**Background and aim:**

Neuropathic pain arises as a direct consequence of a lesion or disease affecting the somatosensory system. Pharmacological treatments for neuropathic pain often fail despite following guidelines. Interdisciplinary Pain Rehabilitation Programs (IPRP) are an effective intervention for chronic pain conditions. Little research has investigated whether IPRP can benefit patients with chronic neuropathic pain compared to other chronic pain conditions.

This study assesses the real-world effects of IPRP on patients with chronic neuropathic pain compared to non-neuropathic patients using Patient-Reported Outcome Measures (PROMs) available in the Swedish Quality Registry for Pain Rehabilitation (SQRP).

**Methods:**

A neuropathic group of patients (*n* = 1,654) were identified in two steps. This group was compared to a non-neuropathic group (*n* = 14,355) composed of common diagnoses (low back pain, fibromyalgia, whiplash associated disorders, and Ehlers-Danlos Syndrome) in relation to background variables, three overall outcome variables, and mandatory outcome variables (pain intensity, psychological distress symptoms, activity/participation aspects and health-related quality of life variables). Of these patients 43–44% participated in IPRP.

**Results:**

At assessment, the neuropathic group reported significantly (with small effect sizes (ES)) more physician visits the previous year, older age, shorter pain durations, and less spatial extent of the pain (moderate ES). Moreover, for the 22 mandatory outcome variables, we found only clinically insignificant differences according to ESs between the groups. For patients participating in IPRP, the neuropathic group displayed equal or in some cases slightly superior results compared to the non-neuropathic group.

**Discussion and conclusion:**

After assessing the real-world effects of IPRP, this large study found that neuropathic pain patients can benefit from the IPRP intervention. Both registry studies and RCTs are needed to better understand which patients with neuropathic pain are most suitable for IPRP and to what extent special considerations need to be made for these patients within the framework of IPRP.

**Supplementary Information:**

The online version contains supplementary material available at 10.1186/s12891-023-06462-2.

## Introduction

Chronic pain has a major impact on health, employment, and daily life. It is associated with considerable personal and economic burdens [1, 2]. Almost half of European adults with chronic pain of moderate to severe intensity report that they have received inadequate pain management [3]. In the International Classification of Diseases (ICD-11), chronic pain includes seven categories of pain conditions that are increasingly being viewed as distinct disease entities rather than just symptoms of something else [4, 5]. One of the seven categories is chronic neuropathic pain (Additional Table 1). Neuropathic pain is defined as pain that arises as a direct consequence of a lesion or disease affecting the somatosensory system [6] and is widely recognized as being one of three major pain mechanisms, along with nociceptive pain and nociplastic pain [7]. About 7% of the population suffers from pain with neuropathic characteristics [8, 9]. In the coming decades, the number of patients with neuropathic pain is likely to increase due to an aging population, an increasing number of cancer survivors suffering from treatment-induced neuropathy [10], and the metabolic syndrome epidemic [11–13] as one in five diabetic patients develops neuropathic pain [14].

**Table 1:** Background data for the two groups at baseline assessment. Note that only a selection of patients later participated in IPRP. For categorial variables, we report the results of Chi^2^ test and for continuous variables t-test for independent groups* denotes significant group difference

Variables	Non-neuropathic group (*n* = 14 355)	Neuropathic group (*n* = 1 654)	Statistics *P*-value	ES
Sex (% men)	22.6	40.6	< 0.001*	NA
Born outside Europe (%)	13.1	14.6	0.082	NA
University education (%)	22.3	22.7	0.702	NA
High healthcare consumption (≥ 4 physician visits; %)	69.3	73.9	< 0.001*	NA
Age (Mean ± SD)	42.8 ± 11.0	45.2 ± 11.0	< 0.001*	-0.22
Not work (days, Mean ± SD)	1393 ± 2592	1431 ± 2742	0.715	-0.02
Pain duration (days, Mean ± SD)	3332 ± 3391	2620 ± 3182	< 0.001*	0.21
Persistent pain duration (days, Mean ± SD)	2656 ± 3015	2136 ± 3125	< 0.001*	0.17
PRI (Mean ± SD)	16.0 ± 9.2	11.3 ± 7.2	< 0.001*	0.53

*ES* effect size (Hedges’ correction), *NA* not applicable, *PRI* Pain Region Index

Whereas management of acute pain focuses on treating the underlying cause, chronic pain management focuses on the effects of the pain, including enhanced functioning and improved quality of life [15]. Systematic Reviews (SRs) have generally reported higher efficacy both at a general level and for specific outcomes of Interdisciplinary Pain Rehabilitation Programs (IPRPs) compared to single-treatment or treatment-as-usual programs (for a review see [16]). A complementary necessary step is to investigate whether the evidence reported in randomized controlled trials (RCTs) and SRs also holds for a consecutive non-selected flow of patients in real-world practice settings. Although the effect sizes according to SRs are moderate at best, there is also practice-based, real-world evidence to support the notion that IPRP is an effective intervention in chronic pain conditions [17–19]. IPRP is based on a biopsychosocial model where pain is viewed as the result of complex and dynamic interactions between physiological, psychological, and social factors [19]. This model contrasts with the traditional biomedical approach, which focuses on purely organic processes related to pain and considers medication and/or surgery as best treatment options. IPRP typically comprises education about pain and coping skills, cognitive behavioural therapy (CBT)-based interventions, and exercise therapy. However, detailed descriptions and recommendations on the content, duration, and intensity of the interventions as well as the indications for referral with respect to the clinical severity of the participants’ chronic pain conditions are scarce [16, 20]. IPRP is provided by a collaborative team of healthcare professionals working closely with the patient to assess and manage pain and its consequences [16]. It is generally offered in chronic pain treatment facilities, with the team structure varying depending on the size of the practice settings, complexity, resources, and patient populations [16, 21].

There are evidence-based guidelines for the pharmacological treatment of neuropathic pain, but even when the guidelines are followed, many patients do not experience adequate pain relief [22]. Nevertheless, compared to many other chronic pain conditions, very little research has been conducted on how IPRP and/or its components may benefit patients with chronic neuropathic pain conditions [23–25]. Hence, the aim of this registry-based cohort study (RBCS) was to assess the real-world effects of IPRP on patients with chronic neuropathic pain compared to chronic non-neuropathic patients (in which IPRP is a more established intervention) using Patient-Reported Outcome Measures (PROMs) available in the Swedish Quality Registry for Pain Rehabilitation (SQRP).

## Methods

### The Swedish Quality Registry for Pain Rehabilitation (SQRP)

The SQRP registers PROMs data from a majority of specialist chronic pain units/departments in Sweden [26]. Patients enrolled in SQRP can be characterised as complex as their health profiles often include psychiatric comorbidities such as depression and anxiety, dysfunctional coping behaviours as well as decreased working life and prolonged sick leave, low participation in social activities, and/or unresponsiveness to routine pharmacological/physiotherapeutic treatments delivered in a monodisciplinary fashion. Strict inclusion and exclusion criteria for inclusion in SQRP are lacking since this is a clinical registry study of patients with complex chronic pain conditions who are mainly referred by primary care to specialist care; a minority of patients are referred from other specialist clinics such as rheumatology and orthopaedic clinical departments. However, the general inclusion criteria for SQRP (and hereby also this study) include disabling chronic pain (on sick leave or experiencing major interference in daily life due to chronic pain), 18 years and older, no further medical examinations required, and written consent to participate. General exclusion criteria for SQRP and this study include severe psychiatric morbidity, abuse of alcohol and/or drugs, diseases that do not allow physical exercise, and specific pain conditions with other evidence-based treatment options available.

PROMs are completed by patients on up to three occasions: before the first visit (baseline assessment) and for those who participate in IPRP immediately after treatment (post-IPRP), and at a 12-month follow-up (12-m fu). The PROMs capture a patient’s background, pain intensity, pain-related cognitions, and psychological distress symptoms as well as activity/participation aspects and health-related quality of life variables.

Due to various clinical or patient-related factors (e.g., need for further clinical examination or assessment, decision to use a unimodal treatment, barriers due to work or transportation, and unwillingness to participate), not all patients assessed at baseline eventually participate in IPRPs.

### Interdisciplinary Pain Rehabilitation Programs (IPRP)

Interdisciplinary Pain Rehabilitation Programs (IPRP) for chronic pain is an interdisciplinary treatment according to the International Association for the Study of Pain (IASP) and is a well-coordinated complex intervention; for detailed descriptions see [16]. Typically, IPRPs are based on cognitive behavioural therapy (CBT) models (including Acceptance Commitment Therapy, ACT) and are administered over several weeks to months [27–30]. The Swedish programs generally include group activities such as supervised physical activity, pain education, training in simulated environments, and CBT coordinated by an interdisciplinary team (e.g., physician, occupational therapist, physiotherapist, psychologist, and social worker) based on a holistic (biopsychosocial) framework [27–30]. The components of IPRP are chosen based on the available evidence for unimodal interventions for chronic pain. The components of IPRP can be active independently or interdependently [31], resulting in a combination of effects explained by known and unknown mechanisms. The effects are assumed to be greater than the sum of its components [32]. The goals of rehabilitation programs for patients with chronic pain [33] are broad and multifactorial in combination with individualised goals of the patient. Complex interventions such as IPRP must have several outcomes measured at multiple levels [34, 35]. Hence, IPRPs are evaluated using many outcomes [28].

### Subjects

Adult patients (i.e., ≥ 18 years) with chronic (≥ 3 months) non-malignant pain registered in the SQRP and assessed between 2008 and 2016 were included. Diagnoses according to the International Classification of Diseases version 10 (ICD-10) are registered in the SQRP. ICD-10 diagnoses that were present in > 0.1% of the patient population of SQRP were reviewed, and 23 of 69 diagnoses were selected as potentially consistent with a neuropathic pain condition (Additional Table 2). Hence, extremely rare ICD-10 diagnoses (i.e., diagnoses present in ≤ 0.1% of our material) were not reviewed. For a patient to be classified as having neuropathic pain, two conditions must be met: the diagnosis is listed in Additional Table 2 and the neuropathic pain mechanism is identified as neuropathic per the physician’s judgement in accordance with SQRP registration routines (these do not specify the use of the NeuPSIG grading system for neuropathic pain [6, 36] but instead rely on the overall clinical judgement of the physician). We did not include Complex Regional Pain Syndrome (CRPS), neither type I nor II [37, 38], in the list of diagnoses presented in Additional Table 2. A total of 1,654 patients with neuropathic pain were identified from 7,046 patients with a diagnosis potentially compatible with neuropathic pain. The control group consisted of 14,355 patients with chronic non-neuropathic pain conditions, namely chronic low back pain (*n* = 6,695), fibromyalgia (*n* = 5,640), Whiplash Associated Disorder (WAD; *n* = 1,226), and Ehlers-Danlos Syndrome including the related Hypermobility Spectrum Disorder (EDS; *n* = 794). No patient in the non-neuropathic group fulfilled the criteria for neuropathic pain. A flow chart is given in Additional Fig. 1.

**Table 2:** Comparisons between those not participating in IPRP and those participating in IPRP for the non-neuropathic group and the Neuropathic group separately. For each diagnostic group, statistics for group differences (*P*-value and ES when significant *P*-value) are listed. Furthest to the right is the two subgroups at baseline receiving IPRP compared (a positive ES indicate higher value in non-neuropathic subgroup)

Group	**Non-neuropathic**								**Neuropathic**								Baseline	
Variables	**No IPRP**			**IPRP**			Statistics		**No IPRP**			**IPRP**			Statistics		Statistics	
Categorial variables	%		n	%		n	P	ES	%		n	%		n	P	ES	P	ES
Sex (% men)	25.0		7986	19.5		6369	< 0.001	NA	45.0		935	34.8		719	< 0.001	NA	< 0.001	NA
Born outside Europe	15.6		7620	10.1		6330	< 0.001	NA	17.4		901	11.1		710	< 0.001	NA	0.381	NA
University education	21.1		7524	23.7		6286	< 0.001	NA	21.1		894	24.8		698	0.085	NA	0.531	NA
Dr visits (> 4)	70.9		7320	67.3		6004	< 0.001	NA	73.5		858	74.2		664	0.407	NA	< 0.001	NA
Continuous variables	Mean	SD	n	Mean	SD	n	p	ES	Mean	SD	n	Mean	SD	n	p	ES	p	ES
Age (years)	42.7	11.4	7986	43.1	10.5	6369	0.033	-0.04	45.8	10.9	935	44.4	11.0	719	0.011	0.13	0.001	-0.13
Not work	1579	2416	3067	1144	2789	2306	< 0.001	0.17	1602	2454	400	1185	3099	277	0.052	-	0.834	-
Pain duration	3373	3355	6718	3284	3432	5786	0.147	-	2834	3192	802	2356	3151	648	0.004	0.15	< 0.001	0.27
Persistent pain duration	2752	2979	5654	2545	3053	4906	< 0.001	0.07	2293	3018	683	1943	3245	558	0.050	0.11	< 0.001	0.20
PRI	15.9	9.6	7986	16.1	8.7	6369	0.252	-	11.2	7.5	935	11.4	6.9	719	0.563	-	< 0.001	0.55
NRS-7 days	7.2	1.8	7502	6.9	1.7	6253	< 0.001	0.16	7.3	1.8	886	7.0	1.7	702	< 0.001	0.22	0.868	-
HADS-A	9.6	5.2	7487	9.2	4.7	6369	< 0.001	0.09	9.4	5.2	888	8.7	4.9	719	0.004	0.14	0.023	0.09
HADS-D	8.9	4.9	7493	8.5	4.4	6362	< 0.001	0.08	9.3	5.0	892	8.4	4.6	719	< 0.001	0.17	0.674	-
MPI-Pain-severity	4.6	0.9	7523	4.4	0.9	6310	< 0.001	0.17	4.6	1.0	892	4.5	0.9	713	0.002	0.16	0.178	-
MPI-Pain-interfer	4.5	1.1	7430	4.4	1.0	6284	< 0.001	0.08	4.5	1.1	880	4.5	1.0	710	0.503	-	0.003	-0.11
MPI-LifeCon	2.6	1.2	7477	2.7	1.1	6299	< 0.001	-0.10	2.6	1.3	888	2.8	1.1	711	0.001	-0.17	0.003	-0.12
MPI-Distress	3.6	1.4	7471	3.5	1.3	6301	< 0.001	0.06	3.6	1.4	887	3.4	1.3	710	0.015	0.12	0.171	-
MPI-Socsupp	4.1	1.4	7447	4.1	1.3	6288	0.253	-	4.4	1.3	887	4.3	1.3	710	0.275	-	< 0.001	-0.17
MPI-punish	1.8	1.5	6788	1.8	1.4	5861	0.009	0.05	1.9	1.4	814	1.7	1.4	667	0.107	-	0.602	-
MPI-protect	3.1	1.5	6750	3.0	1.4	5840	< 0.001	0.07	3.2	1.4	806	3.1	1.4	662	0.047	0.10	0.060	-
MPI-distract	2.6	1.3	6782	2.5	1.2	5857	0.164	-	2.7	1.3	810	2.6	1.2	665	0.019	0.12	0.590	-
MPI-GAI	2.3	0.9	7487	2.5	0.8	6304	< 0.001	-0.19	2.2	1.0	890	2.4	0.9	709	< 0.001	-0.22	0.002	0.13
EQ-5D-index	0.2	0.3	7325	0.3	0.3	6028	< 0.001	-0.13	0.2	0.3	866	0.2	0.3	664	0.027	-0.11	< 0.001	0.19
EQ-VAS	39.7	20.9	7158	41.1	18.9	5981	< 0.001	-0.07	37.9	21.3	834	40.2	19.6	651	0.037	-0.11	0.246	-
sf36-pf	48.8	22.4	7408	52.5	20.0	6130	< 0.001	-0.17	45.2	22.7	868	48.9	19.8	677	< 0.001	-0.17	< 0.001	0.18
sf36-rp	13.1	25.6	7223	12.4	24.0	6092	0.107	-	12.8	26.3	850	11.6	23.4	666	0.361	-	0.397	-
sf36-bp	22.4	15.0	7418	24.0	13.8	6133	< 0.001	-0.11	20.9	15.1	873	22.2	14.0	677	0.097	-	0.001	0.13
sf36-gh	37.6	21.4	7291	40.8	20.4	6087	< 0.001	-0.16	40.6	21.7	856	43.9	20.2	668	0.003	-0.16	< 0.001	-0.15
sf36-vt	22.8	19.3	7388	22.7	18.2	6127	0.751	-	25.5	20.0	863	26.1	19.1	677	0.564	-	< 0.001	-0.19
sf36-sf	45.8	26.8	7403	47.1	25.1	6133	0.004	-0.05	44.6	27.4	872	46.6	26.8	676	0.148	-	0.647	-
sf36-re	41.3	43.5	7120	42.2	42.8	6044	0.220	-	37.5	43.6	828	43.6	43.7	657	0.008	-0.14	0.452	-
sf36-mh	53.3	23.3	7375	55.0	21.2	6123	< 0.001	-0.07	51.6	24.4	861	56.6	21.6	677	< 0.001	-0.22	0.058	-

*ES* effect size (Hedges’ correction for between groups), *NRS-7 days* Pain intensity as measured by a numeric rating scale for the previous 7 days, *HADS* Hospital Anxiety and Depression Scale, *MPI* Multidimensional Pain Inventory; *EQ-5D-index* The index of the European quality of life instrument, *EQ-VAS* The European quality of life instrument thermometer-like scale, *sf36* The Short Form (36) Health Survey. See Methods for explanations of the subscale abbreviations

### Patient-Reported Outcome Measures (PROMs)

#### Background variables

As described in detail elsewhere [39–42] the following background data were retrieved from the registry:

- age (years)
- sex
- education level (university versus no university)
- country of birth (born in versus outside of Europe)
- number of physician visits due to pain during the last year (categories: 0–1 times, 2–3 times and ≥ 4 times); ≥ 4 times indicates high healthcare consumption
- days with no work or studies
- pain duration (days)
- spatial extent of pain (i.e., spreading of pain) was quantified by counting the number of reported painful areas using 36 predefined anatomical regions [18, 42] this variable was denoted as Pain Region Index (PRI).

### The 22 mandatory outcome variables in SQRP

There are 22 mandatory outcome variables in the SQRP registered on up to three occasions (baseline assessment, post-IPRP, and at 12-m fu). Psychometric properties have been presented elsewhere [18, 43–46]. Predefined rules are used to handle single missing items of a scale or a subscale in SQRP [47]. The mandatory outcome variables are in good agreement with the biopsychosocial model of chronic pain and the outcome domains presented by the Initiative on Methods, Measurement, and Pain Assessment in Clinical Trials (IMMPACT) [43, 48] and the Validation and Application of a patient-relevant core set of outcome domains to assess multimodal PAIN therapy (VAPAIN) [33] initiatives. The 22 mandatory variables have been detailed elsewhere [39–42], so they are only briefly described below:

- Mean pain intensity over the past seven days is measured using an 11-point numerical rating scale (0 = no pain to 10 = worst possible pain; NRS-7 days).
- Psychological distress is measured using the two subscales of the Hospital Anxiety and Depression Scale (HADS), which capture signs of depression (HADS-D) and anxiety (HADS-A) [49, 50].
- The Swedish version of the Multidimensional Pain Inventory (MPI) is used to describe the multidimensional nature of chronic pain. Part one is composed of five subscales: pain severity (MPI-Pain-severity), pain-related interference (MPI-Pain-interfer), life control (MPI-LifeCon), psychological distress (MPI-Distress), and perceived social support (MPI-Socsupp). In part two, patients report how they perceive significant others’ responses to pain or suffering expressed by the patient in three subscales: punishing responses (MPI-punish), solicitous responses (MPI-protect), and distracting responses (MPI-distract). Part three measures participation in various activities, which are combined into a General Activity Index (MPI-GAI) [51].
- Perceived health is measured using the European Quality of Life instrument (EQ-5D) [52–54]. This instrument comprises two variables. The first is an index (EQ-5D-index) based on five dimensions: mobility, self-care, usual activities, pain/discomfort, and anxiety/depression. The second measures current perceived health according to a thermometer-like 100-point scale (EQ-VAS) with defined end points (high values indicate good health and low values indicate poor health).
- The Short Form Health Survey (sf36) assesses eight health aspects/dimensions (scored from 0 to 100, with higher scores indicating a better perception of health) [55]: 1) physical functioning (sf36-pf); 2) role limitations due to physical functioning (sf36-rp); 3) bodily pain (sf36-bp); 4) general health (sf36-gh); 5) vitality (sf36-vt); 6) social functioning (sf36-sf); 7) role limitations due to emotional problems (sf36-re); and 8) mental health (sf36-mh).

### Overall outcome measures

The overall effect of IPRP over time was measured using the Multivariate Improvement Score (MIS), which has been recently described and used in several SQRP studies [39–42]. In short, MIS is a comprehensive relative measure of changes across 18 of the 22 mandatory variables; the changes in the 18 variables were significantly correlated according to an advanced principal components analysis at post-IPRP and at 12-m fu [42]. Changes in MPI-Socsupp, MPI-punish, MPI-distract and MPI-protect did not significantly correlate with changes in the other 18 mandatory variables according to the same analysis and therefore were not included in MIS. Higher MIS indicates a larger overall relative improvement following IPRP [42].

Additionally, at post-IPRP and at 12-m fu, patients retrospectively estimated the degree of positive change in pain (*Change-pain*) and in their ability to cope with life situations (*Change-life situation*). The *Change-pain* item was rated on a five-point Likert scale: markedly increased pain (0), somewhat increased pain (1), no change in pain (2), somewhat diminished pain (3), and markedly diminished pain (4). *Change-pain* was trichotomized into increased pain (i.e., markedly increased pain (0) and somewhat increased pain (1)), no change (2), and diminished pain (i.e., somewhat diminished pain (3), and markedly diminished pain (4)).

The *Change-life situation* item was rated on a five-point Likert scale: markedly worsened (0), somewhat worsened (1), no change (2), somewhat improved (3), and markedly improved (4). *Change-life situation* was trichotomized into worsened (i.e., markedly worsened (0), and somewhat worsened (1)), no change (2), and improved (i.e., somewhat improved (3) and markedly improved (4)). In the text and tables, the proportion of patients who report ‘diminished pain’ and ‘improved’ is given for *Change-pain* and *Change-life situation*, respectively.

### Statistics

The statistical package IBM SPSS Statistics (version 28.0; IBM Corporation, Route 100 Somers, New York, USA) was used. In the text and tables, the mean value ± one standard deviation (± 1 SD) of continuous variables and percentages (%) for categorical variables are reported. For analysis of within group changes, Student’s t-test for paired observations was used. To compare groups, Student’s t-test for independent samples and Chi square test were applied. In large samples, small differences may be significant and effects sizes can be used to evaluate the clinical importance of the significant differences. Effect sizes (ES; Cohen’s d) for within group analyses were computed and Hedges’ correction – a measure of effect size weighted by the relative size of each sample – was used for between group ES. The absolute effect size was considered clinically insignificant for < 0.20, small for 0.20–0.49, moderate for 0.50–0.79, and large for ≥ 0.80 [56].

## Results

### Baseline assessments -total groups

#### Background data

The proportion of men was significantly higher in the neuropathic group than in the non-neuropathic group (41% vs. 23%, *p* < 0.001). The neuropathic group reported significantly more physician visits the previous year, higher age, shorter pain durations, and less spatial extent of pain according to PRI (Table 1). Effect sizes (ESs) were small, except for PRI, which was associated with a moderate ES.

### The 22 outcome variables

Of the 22 mandatory outcome variables, 13 differed significantly between the two groups at baseline assessment (Additional Table 3). However, these statistically significant differences were not clinically significant according to the magnitude of the ESs. Furthermore, no consistent pattern could be discerned between the two patient groups (Additional Table 3).

**Table 3:** Overall effects of IPRP in the two groups according to the Multivariate Improvement Score (MIS) post-IPRP and at 12-month follow-up. * denotes significant group difference

**Variables**	**Non-neuropathic pain group** Mean ± SD	n	**Neuropathic pain group** Mean ± SD	n	**Statistics** *P*-value	**ES**
MIS post IPRP	0.00 ± 2.50	6 348	0.03 ± 2.73	715	0.809	-0.01
MIS 12-m fu	-0.08 ± 2.69	3 828	0.23 ± 2.96	544	0.014*	-0.11

*ES* effect size (Hedges’ correction), *12-m fu* 12-month follow-up

### Participation in IPRP

Of the non-neuropathic patients, 44% took part in IPRP; the corresponding figure for neuropathic pain patients was 43%. In both groups, those who participated in IPRP generally had a slightly better clinical presentation than those who did not participate in IPRP (Table 2). Importantly, among statistically significant results, only three variables in the neuropathic group barely reached the small ES level (0.20–0.49) (Table 2); all variables in the non-neuropathic group were insignificant according to the magnitude of the ESs.

The higher female proportion increased further for those participating in IPRP. In the neuropathic group, 59% were women at assessment (baseline) and in the subgroup participating in IPRP 65% were women (Tables 1 and 2). Corresponding figures for the non-neuropathic group were 77% and 80%. For those participating in IPRP the neuropathic group had significantly higher health consumption previous year compared to the non-neuropathic group (Table 2). For the continuous variables displayed in Table 2 the two groups participating in IPRP showed a few significant differences, but these were generally associated with clinically insignificant effect sizes (i.e. < 0.20). The exceptions were the spatial extent of pain (PRI) which was higher in the non-neuropathic group (moderate effect size). Pain durations were also significantly longer in the non-neuropathic group (small effect sizes).

### Overall outcome variables after IPRP

#### Multivariate Improvement score (MIS)

No significant group difference in MIS existed post-IPRP (Table 3). At the 12-m fu, a significant group difference with better results for the neuropathic pain group was noted but the ES was insignificant (i.e., < 0.20) (Table 3).

### Change-pain and Change-life situation

Significantly higher improvements for *Change-pain* were noted for the neuropathic pain group (61.8–63.0%) compared to the non-neuropathic group (56.2–56.4%) at both time points (Table 4)*.*

**Table 4:** Change in pain and change in life situation post-IPRP and at 12-month follow-up. The right side shows the statistics. * denotes significant group difference

Improved in:	**Non-neuropathic pain group** %	**Neuropathic pain group** %	**Statistics** *P*-value
Change pain post IPRP	56.4	61.8	0.018*
Change pain 12-m fu	56.2	63.0	0.012*
Change life situation post IPRP	84.5	83.8	0.303
Change life situation 12-m fu	77.2	79.2	0.406

*12-m fu* 12-month follow-up

For the ability to handle life situations in general (i.e., *Change-life situation*), we found no significant group differences at the two time points (Table 4). Prominent proportions of both cohorts reported improvements (> 75%).

### Changes in the 22 mandatory outcome variables after IPRP

#### Within group changes

When comparing baseline assessment and post-IPRP, both groups displayed significant improvements in all mandatory outcome variables except for MPI-punish and MPI-distract (Table 5). Most ESs were small, but some of the variables were associated with moderate ESs (Table 5). Hence, both groups displayed moderate ESs for MPI-pain severity, sf36 bodily pain, and sf36 vitality. In the non-neuropathic group, MPI-LifeCon was also associated with a moderate ES, whereas EQ-5D-index and MPI-pain interference were associated with moderate ESs in the neuropathic group.

**Table 5:** The differences between baseline assessment and post-IPRP (a positive value indicates improvement) for the 22 outcome variables in the two groups. Both within group and between group statistics (*p*-value and effect sizes) are shown. * denotes significant group difference

	**Non-neuropathic group**		Statistics		**Neuropathic group**		Statistics		Statistics	
	Mean	SD	Within	group	Mean	SD	Within group		Between groups	
Variables			*P*-value	ES			*P*-value	ES	*P*-value	ES
diff-NRS-7days^a^	0.88	1.99	< .001	0.44	1.01	2.16	< .001	0.47	0.100	-0.07
diff-HADS-A^a^	1.30	3.86	< .001	0.34	1.10	3.91	< .001	0.28	0.184	0.05
diff-HADS-D^a^	1.87	3.79	< .001	0.49	1.54	3.96	< .001	0.39	0.029*	0.09
diff-MPI-Pain-severity^a^	0.50	0.95	< .001	0.53	0.57	1.04	< .001	0.55	0.056	-0.08
diff-MPI-Pain-interfer^a^	0.43	0.88	< .001	0.49	0.49	0.95	< .001	0.52	0.093	-0.07
diff-MPI-LifeCon	0.61	1.21	< .001	-0.50	0.50	1.26	< .001	-0.40	0.019*	0.09
diff-MPI-distress^a^	0.60	1.35	< .001	0.44	0.54	1.32	< .001	0.41	0.261	0.04
diff-MPI-Socsupp	-0.19	1.01	< .001	0.19	-0.30	1.03	< .001	0.29	0.005*	0.11
diff-MPI-punish^a^	0.02	1.15	0.143	0.02	-0.01	1.17	0.871	-0.01	0.532	0.03
diff-MPI-protect	-0.13	1.05	< .001	0.12	-0.20	1.08	< .001	0.19	0.104	0.07
diff-MPI-distract	0.02	0.99	0.065	-0.03	-0.04	1.03	0.370	0.04	0.141	0.06
diff-MPI-GAI	0.18	0.70	< .001	-0.26	0.24	0.80	< .001	-0.31	0.021*	-0.09
diff-EQ-5D-index	0.12	0.33	< .001	-0.38	0.20	0.35	< .001	-0.56	< .001*	-0.21
diff-EQ-VAS	9.95	21.71	< .001	-0.46	10.44	23.06	< .001	-0.45	0.589	-0.02
diff-sf36-pf	4.65	15.63	< .001	-0.30	6.49	18.32	< .001	-0.35	0.004*	-0.12
diff-sf36-rp	9.75	33.19	< .001	-0.29	10.51	35.02	< .001	-0.30	0.580	-0.02
diff-sf36-bp	8.46	16.27	< .001	-0.52	10.04	17.15	< .001	-0.59	0.017*	-0.10
diff-sf36-gh	4.91	17.00	< .001	-0.29	4.01	16.91	< .001	-0.24	0.193	0.05
diff-sf36-vt	12.26	21.59	< .001	-0.57	11.46	22.20	< .001	-0.52	0.361	0.04
diff-sf36-sf	7.64	24.94	< .001	-0.31	8.31	24.42	< .001	-0.34	0.501	-0.03
diff-sf36-re	8.79	46.38	< .001	-0.19	5.30	47.11	0.005	-0.11	0.072	0.08
diff-sf36-mh	7.71	19.84	< .001	-0.39	6.19	19.78	< .001	-0.31	0.058	0.08

*diff* change in a certain variable (generally post IPRP – pre but for variables marked with ^a^ pre-post IPRP), *ES* effect size (Hedges’ correction for between groups and Cohen’s d for within group), *NRS-7 days* Pain intensity as measured by a numeric rating scale for the previous 7 days, *HADS* Hospital Anxiety and Depression Scale, *MPI* Multidimensional Pain Inventory, *EQ-5D-index* The index of the European quality of life instrument, *EQ-VAS* The European quality of life instrument thermometer-like scale, *sf36* The Short Form (36) Health Survey. See Methods for explanations of the subscale abbreviations

At the 12-m fu, 21 of the 22 mandatory outcomes showed significant within-group changes in both groups (Table 6); the exception in both groups was MPI-punish. Again, most ESs were small. However, MPI-pain severity, MPI-pain interference, and sf36 bodily pain were associated with moderate ESs in both groups. In the neuropathic group, NRS-7d and the two EQ-5D variables also had moderate ESs (Table 6).

**Table 6:** The differences between baseline assessment and 12-month follow-up (a positive value indicates improvement) for the 22 outcome variables in the two groups. Both within group and between group statistics (*p*-value and effect sizes) are shown. * denotes significant group difference

	**Non-neuropathic group**		Statistics		**Neuropathic group**		Statistics		Statistics	
	Mean	SD	Within	group	Mean	SD	Within group		Between groups	
Variables			*P*-value	ES			*P*-value	ES	*P*-value	ES
diff-NRS-7days^a^	0.99	2.15	< .001	0.46	1.23	2.30	< .001	0.53	0.018*	-0.11
diff-HADS-A^a^	1.36	4.01	< .001	0.34	1.42	4.49	< .001	0.32	0.723	-0.02
diff-HADS-D^a^	1.46	4.01	< .001	0.37	1.55	4.22	< .001	0.37	0.634	-0.02
diff-MPI-Pain-severity^a^	0.61	1.11	< .001	0.55	0.78	1.21	< .001	0.65	< .001*	-0.15
diff-MPI-Pain-interfer^a^	0.57	1.09	< .001	0.53	0.70	1.20	< .001	0.59	0.012*	-0.12
diff-MPI-LifeCon	0.53	1.26	< .001	-0.42	0.49	1.34	< .001	-0.37	0.564	0.03
diff-MPI-distress^a^	0.48	1.39	< .001	0.35	0.56	1.39	< .001	0.41	0.213	-0.06
diff-MPI-Socsupp	-0.39	1.16	< .001	0.33	-0.50	1.15	< .001	0.44	0.031*	0.10
diff-MPI-punish^a^	0.02	1.21	0.424	0.01	0.00	1.28	0.998	0.00	0.782	0.01
diff-MPI-protect	-0.19	1.19	< .001	0.16	-0.31	1.15	< .001	0.27	0.032*	0.11
diff-MPI-distract	-0.06	1.07	0.001	0.06	-0.17	1.02	< .001	0.17	0.031*	0.11
diff-MPI-GAI	0.14	0.78	< .001	-0.18	0.26	0.91	< .001	-0.28	0.002*	-0.14
diff-EQ-5D-index	0.16	0.35	< .001	-0.44	0.25	0.37	< .001	-0.68	< .001*	-0.27
diff-EQ-VAS	10.48	23.68	< .001	-0.44	14.03	24.91	< .001	-0.56	0.001*	-0.15
diff-sf36-pf	6.00	18.16	< .001	-0.33	8.03	19.52	< .001	-0.41	0.018*	-0.11
diff-sf36-rp	14.08	37.06	< .001	-0.38	15.02	40.15	< .001	-0.37	0.595	-0.03
diff-sf36-bp	10.23	18.46	< .001	-0.55	13.72	19.91	< .001	-0.69	< .001*	-0.19
diff-sf36-gh	4.58	19.22	< .001	-0.24	3.69	18.40	< .001	-0.20	0.330	0.05
diff-sf36-vt	9.45	22.38	< .001	-0.42	10.27	23.56	< .001	-0.44	0.443	-0.04
diff-sf36-sf	8.67	26.47	< .001	-0.33	9.04	27.02	< .001	-0.34	0.765	-0.01
diff-sf36-re	10.99	48.67	< .001	-0.23	9.88	52.56	< .001	-0.19	0.640	0.02
diff-sf36-mh	6.54	21.07	< .001	-0.31	5.68	21.56	< .001	-0.26	0.390	0.04

*diff* Change in a certain variable (generally post IPRP – pre but for variables marked with ^a^ pre-post IPRP), *ES* Effect size (Hedges’ correction for between groups and Cohen’s d for within group, *NRS-7 days* Pain intensity as measured by a numeric rating scale for the previous 7 days, *HADS* Hospital Anxiety and Depression Scale, *MPI* Multidimensional Pain Inventory, *EQ-5D-index* The index of the European quality of life instrument, *EQ-VAS* The European quality of life instrument thermometer-like scale, *sf36* The Short Form (36) Health Survey. See Methods for explanations of the subscale abbreviations

### Between group comparisons

Seven of the 22 mandatory variables displayed significant differences in changes (baseline assessment vs. post-IPRP) when comparing the non-neuropathic group and the neuropathic group (Table 5). Five of these showed better results for the neuropathic group and two for the non-neuropathic group. However, according to the effect sizes, all variables except EQ-5D-index (small ES) were associated with insignificant ESs.

After 12 months, 11 of the 22 mandatory outcome variables showed significant differences in changes (baseline assessment vs. 12-m fu) when comparing the non-neuropathic group and the neuropathic group (Table 6). All eleven differences in changes displayed better results for the neuropathic group. EQ-5D-index had a small ES whereas the other significant variables were associated with insignificant ESs.

## Discussion

### Major results

From a personalized medicine point of view, it is important to ascertain the effect of interventions in different subgroups of chronic pain patients. To our knowledge, this large-scale study is the first study to find that IPRP’s effectiveness treating neuropathic pain is equal to or in some cases slightly superior to treating non-neuropathic chronic pain conditions. Baseline assessments – both of the total cohorts and of those participating in IPRP—displayed some differences in background profiles, but the clinical severity did not differ in ESs according to a broad biopsychosocial coverage using the 22 mandatory variables.

### Overall outcomes variables

This is the first study to look at the real-world effects of comprehensive IPRP on patients with chronic neuropathic pain compared to non-neuropathic patients. The latter group consisted mainly of low back pain and fibromyalgia; presumably, most non-neuropathic patients would therefore likely be classified as nociplastic with today’s classification [7]. For neuropathic pain conditions, guidelines have traditionally been focused on unimodal treatments such as pharmacological treatments and physical therapy delivered separately [24]. The focus has increasingly shifted to recommendations that include biopsychosocial aspects (i.e., well-integrated interdisciplinary rehabilitation programs) [24, 57]. However, the evidence for this approach, particularly in chronic neuropathic pain conditions, has been sparse and appears to be based on the fact that IPRPs were not designed for specific diagnoses. Since chronic neuropathic pain has traditionally been considered difficult to treat and patient satisfaction with therapy has been low [58], our results are encouraging. Our overall outcome variables did not show prominent differences between the two patient groups, but the differences noted (i.e., MIS at 12-m fu and change-pain at both time points) indicated slightly better results in the neuropathic group.

A RBCS from SQRP reports that women had to some extent better outcomes than men on MIS and the two retrospective items (i.e., Change-pain and Change-life situation) [39]. Hence, speculatively a similar sex proportion may have increased the difference in favour of the neuropathic group. Educational level and country of birth were also associated with differences in that study [39], but these factors did not differ between the present two groups neither for the total cohorts nor for those participating in IPRP.

### Outcomes for the 22 mandatory variables

Shaygan et al. demonstrated the beneficial effects of an inpatient multidisciplinary program for neuropathic pain [25]. However, the participants were from a single clinical department and the follow-up was only 3 months. Our within-group analyses confirm these results for both the neuropathic and the non-neuropathic groups in a larger sample size and with longer follow-up (Tables 5 and 6). For the total registry (regardless of chronic pain diagnoses), we have recently reported small to moderate effects on the 22 mandatory outcomes and the findings reported here for both groups provided additional support for the former [42]. In both groups, prominent proportions of the 22 mandatory variables had improved significantly at both time points as specified by the within-group analyses (Table 5 and 6). For example, at 12-m fu, pain intensity (NRS-7 days, MPI-Pain-severity and sf-36 bp), pain interference (MPI-Pain-interfer), and health aspects (EQ-5D-index and EQ-VAS) in the neuropathic group were associated with moderate ESs, and the other significant variables were associated with small ESs. A similar pattern, despite some differences in the magnitudes of ESs, was also found in the non-neuropathic group. It is also important to note that the significant pain intensity effects from both our study and Shaygan et al.’s study contrast with some systematic reviews that report no evidence of efficacy in relation to this outcome [10, 11]. As Swedish specialist care IPRPs have largely adopted the idea of acceptance as the cornerstone of the psychological component of IPRP, patients are discouraged from setting pain reduction goals. It is arguably ethically problematic to disregard the wishes of the patients regarding pain intensity, and we may also underestimate patients’ ability to grasp the facets of chronic pain and its maintenance factors [42].

The between group analyses of changes in the 22 mandatory outcomes showed that the significant differences were in favour of the neuropathic group (five of seven post-IPRP and all 11 at 12-m fu) (Tables 5 and 6). However, all – except for EQ-5D-index—significant differences were insignificant based on the applied criteria used for ESs. In other words, IPRP was at least as effective for the neuropathic group as for the non-neuropathic group for the 22 mandatory outcomes.

Despite the improvements seen in both groups for the absolute majorities of the mandatory variables, MPI-punish did not change in any group. Although the usual contents of IPRPs in Sweden involve dialogue with participants’ families, these results may indicate the need for further research and development of the IPRP programs in this regard.

### Referral patterns and Baseline differences

Most patients with chronic pain in Sweden are managed in primary care and only a few are referred to specialist care [3, 15]. Although there are national guidelines on when it is appropriate to refer patients with chronic pain to specialized clinics for biopsychosocial assessment and eventually IPRP, compliance with these guidelines is currently non-transparent [59]. Decision-making in management of pain is a complex process and may be influenced by both patient and physician [60]. Pain management choices are often unsystematic, dependent on nonmedical factors, and vary across demographic groups [61]. The proportion of patients with neuropathic pain conditions in SQRP is relatively small compared to population numbers, which may reflect certain assumptions about the benefits of biopsychosocial assessments and possibly IPRP for these patients.

The proportion of men was significantly higher in the neuropathic group (Tables 1 and 2). Epidemiological studies indicate a higher female prevalence of musculoskeletal chronic pain conditions and pain conditions with neuropathic characteristics in the population, although not with such pronounced sex differences as in the present two groups [8, 62–64]. The reasons for this skewed assortment/selection are unclear and need to be investigated. The question arises whether the larger proportion of men in the neuropathic group influenced results; however, a recent large SQRP study could not confirm other reports that women assessed at specialist clinics reported a more severe clinical situation than men or are more prone to behavioural changes [39].

The association between baseline pain duration and clinical outcomes is important when pain duration is less than 12 months [65]; the duration of pain in this study was on average ≥ 7 years in both groups. The non-neuropathic pain group reported a significantly longer pain duration and was associated with a small ES despite being slightly younger than the neuropathic pain group. The non-neuropathic pain patients reported that they developed persistent pain after approximately two years, compared to one year in the neuropathic group. This finding in the neuropathic group may indicate another trajectory for the development of a complex pain condition. For example, neural activity has been found to change at multiple levels of the ascending pain pathway in patients with neuropathic pain [66]. This finding may also explain the higher healthcare consumption in the neuropathic group. However, the higher healthcare consumption could also be the result of different treatment strategies in the two groups as the neuropathic group typically receives more pharmacological interventions and evaluations of treatment effects before referral to the specialist clinics.

Chronic musculoskeletal pain is considered a continuum of ‘‘widespreadness” from localized pain to more generalized pain related to the progression of peripheral and central sensitization [67–69]. Patients with neuropathic pain reported less spreading of pain compared to those with non-neuropathic pain. However, there was no difference regarding spreading of pain between those who participated in IPRP and those who did not in either of the groups (Table 2). A previous cohort study from the SQRP found that more widespread pain was associated with a longer pain duration and a more severe clinical picture at baseline [39]. Studies investigating spreading of pain are mostly focused on musculoskeletal pain and/or do not distinguish between neuropathic and non-neuropathic pain. Thus, using spreading of pain as an indicator of severity might be misleading for chronic neuropathic pain.

At the baseline assessment of the total cohorts (Additional Table 3), most of the significant differences in the 22 mandatory variables indicated a more severe situation for the neuropathic group. Against such an interpretation, it could be argued that all ESs were insignificant (Additional Table 3); a similar situation with insignificant ESs was found for those participating in IPRP (Table 2). Hence, patients with neuropathic pain conditions who were referred to the Swedish specialist clinics and included in the SQRP had a similar level of severity as the non-neuropathic group. However, this does not exclude that subjects with chronic neuropathic pain conditions in the population have a more severe clinical presentation than subjects with non-neuropathic pain conditions [70].

### Participation and Patient selection

The proportion of patients included in IPRP after assessment was similar for both the neuropathic and non-neuropathic groups (43% and 44%, respectively), indicating that the assessments about the potential benefits of IRPPs were not primarily influenced by pain mechanistic descriptions.

Those who participated in IPRP generally had slightly better clinical presentations than those who did not participate in IPRP (Table 2) in both groups, but ESs were generally insignificant. This finding is in line with previous research demonstrating that patients with a more severe clinical presentation participated somewhat less in IPRP, but those who did showed the largest improvements in outcomes [18]. More research is needed to understand why patients with more severe clinical presentation are not enrolled and/or do not participate in the IPRP.

We also noted that the female dominance increased further for those participating in IPRP. The reasons for this are unclear and further investigations are warranted. The components of IPRPs and the manner they are presented may be unequally appeal for the two sexes. Most of the team members responsible for the IPRPs in Sweden are women is another factor that may have contributed. The selection process is an interaction between assessment teams and patients in which communication skills, willingness to participate in IPRP, conceptions about IPRP and past experiences might play important roles, that are not included in the data captured by the registry.

### Strengths and limitations

The present RBCS cannot provide the same level of evidence as RCTs. There is no control group not receiving IPRP, so we cannot rule out an improvement caused by unknown factors. However, given the high proportion of specialty-level pain clinics providing data to SQRP and the high number of chronic pain patients included, the present study can provide generalizable evidence. As such, referral bias is minimized by a naturalistic, more realistic selection of patients, which also offers advantages related to generalizability [71]. The limitations of disease registries can also be minimized through rigorous database design and data collection. Therefore, it cannot be ruled out that there was a higher degree of selection for referral to specialist pain management in the neuropathic group than in the non-neuropathic group. As discussed elsewhere, although validated PROM instruments have been used, these could be problematic in repeated evaluations related to treatments/interventions [41, 42, 72]. Retrospective items such as *Change-pain* and *Change-life* situation may be also problematic [73–75]. However, MIS and these two items generally showed the same pattern when the two patient groups were compared. We have no reason to suspect that these potential PROM limitations were more pronounced in either group. Objective outcomes such as sick leave or work participation were not included, although such measures are also subject to limitations. As there is an ongoing debate about whether CRPS (type I and/or II) displays neuropathic characteristics, it was not included in any of the two groups [27, 28]. However, the low number of patients with these diagnoses (total *n* = 3) could not have biased our results. Finally, the NeuPSIG grading system for neuropathic pain [6, 36] had not been implemented in SQRP; we acknowledge that as a limitation.

## Conclusions

This large study assessed the real-world effects of IPRP on patients with chronic neuropathic pain compared to non-neuropathic patients using PROM data from the SQRP. Our results show that IPRP yields equal or in some cases slightly superior outcomes for neuropathic pain conditions compared to non-neuropathic chronic pain conditions. In the neuropathic pain group, the proportion of men was higher and the duration of chronic pain shorter, but persistent pain appeared relatively earlier in the pain processes. According to the variables used for the IPRP results, there were no other clinically relevant differences between the two patient groups at baseline. Most importantly, however, this study recommends that patients with neuropathic pain be offered IPRP.

## Supplementary Information

**Additional Figure.**

**Additional Tables.**

## Data Availability

The datasets generated and/or analysed in this study will be available upon reasonable request from the corresponding author or from SQRP.

## Abbreviations

12-m fu: 12-Month follow-up
ACT: Acceptance Commitment Therapy
CBT: Cognitive behavioural therapy
Change-life situation: Retrospectively estimated change in ability to cope with life situations
Change-pain: Retrospectively estimated degree of positive change in pain
CRPS: Complex Regional Pain Syndrome
EDS: Ehlers-Danlos Syndrome
EQ-5D: European Quality of Life instrument
EQ-5D-index: Index of EQ-5D
EQ-VAS: Perceived health scale of EQ-5D
ES: Effect size
HADS: Hospital Anxiety and Depression Scale
HADS-D: Depression subscale of HADS
HADS-A: Anxiety subscale of HADS
IASP: International Association for the Study of Pain
ICD: The International Classification of Diseases
IMMPACT: Initiative on Methods, Measurement, and Pain Assessment in Clinical Trials
IPRP: Interdisciplinary Pain Rehabilitation Program
MIS: Multivariate Improvement Score
MPI: Multidimensional Pain Inventory
MPI-Pain-severity: Pain severity subscale of MPI
MPI-Pain-interfer: Pain-related interference subscale of MPI
MPI-LifeCon: Life control subscale of MPI
MPI-Distress: Psychological distress subscale of MPI
MPI-Socsupp: Perceived social support subscale of MPI
MPI-punish: Punishing responses subscale of MPI
MPI-protect: Solicitous responses subscale of MPI
MPI-distract: Distracting responses subscale of MPI
MPI-GAI: General Activity Index subscale of MPI
NeuPSIG: IASP Neuropathic Pain Special Interest Group
NRS-7 days: Mean pain intensity over the past seven days
post-IPRP: IPRP immediately after treatment
PRI: Pain Region Index
PROM: Patient-Reported Outcome Measure
RBCS: Registry-based cohort study
RCT: Randomized controlled trial
sf36: The Short Form Health Survey
sf36-pf: Physical functioning—subscale of sf36
sf36-rp: Role limitations due to physical functioning- subscale of sf36
sf36-bp: Bodily pain—subscale of sf36
sf36-gh: General health—subscale of sf36
sf36-vt: Vitality—subscale of sf36
sf36-sf: Social functioning—subscale of sf36
sf36-re: Role limitations due to emotional problems—subscale of sf36
sf36-mh: Mental health—subscale of sf36
SQRP: Swedish Quality Registry for Pain Rehabilitation
SR: Systematic Review
WAD: Whiplash Associated Disorder
VAPAIN: Validation and Application of a patient-relevant core set of outcome domains to assess multimodal PAIN therapy
