# Supplementary material for: Effects of interdisciplinary pain rehabilitation programs on neuropathic and non-neuropathic chronic pain conditions – a registry-based cohort study from Swedish Quality Registry for Pain Rehabilitation (SQRP)
Source: BMC Musculoskelet Disord. 2023 May 6;24:357. doi: 10.1186/s12891-023-06462-2 (PMC10163768; doi:10.1186/s12891-023-06462-2)
Supplement: Supplementary file 1 — Additional Figure. [file 12891_2023_6462_MOESM1_ESM.docx]

**Additional Figure 1**

| 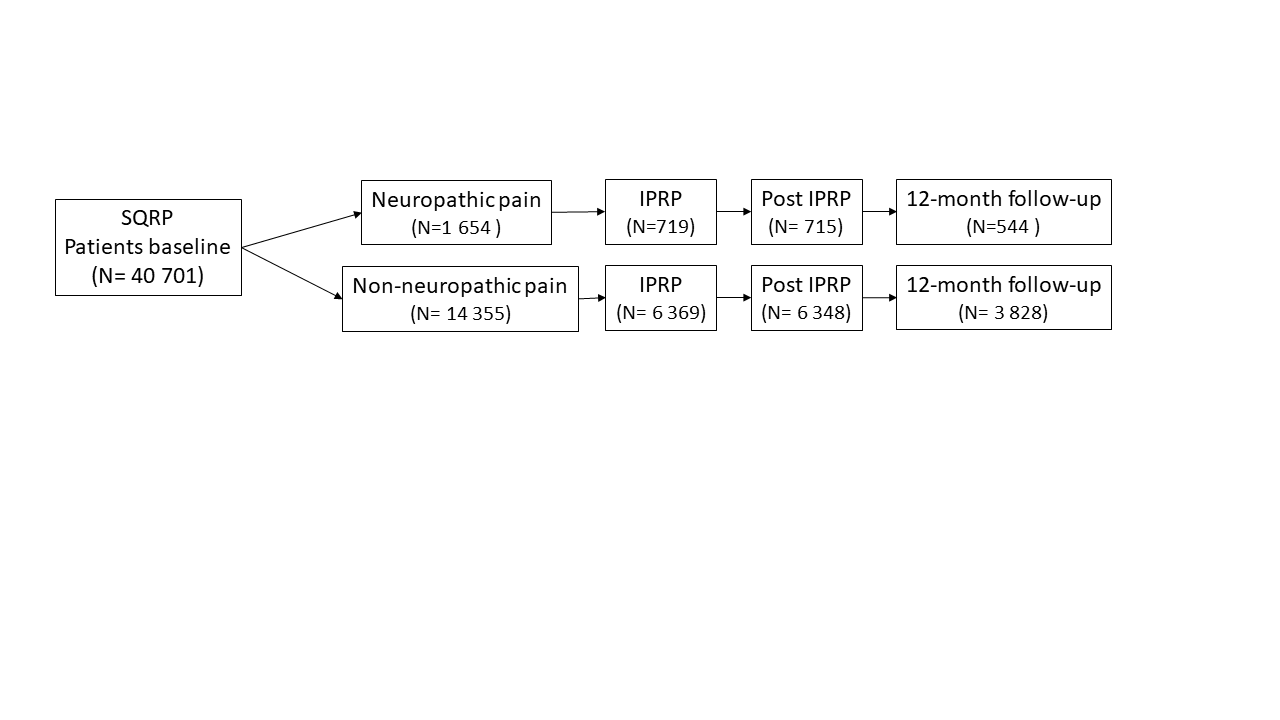 |
| --- |
| **Additional Figure 1**: Flow chart. The non-neuropathic group consisted of the following diagnoses: low back pain, fibromyalgia, whiplash associated disorders, and Ehlers-Danlos Syndrome. SQRP: Swedish Quality Registry for Pain Rehabilitation; IPRP: Interdisciplinary Pain Rehabilitation Program |
