# Supplementary material for: Effects of interdisciplinary pain rehabilitation programs on neuropathic and non-neuropathic chronic pain conditions – a registry-based cohort study from Swedish Quality Registry for Pain Rehabilitation (SQRP)
Source: BMC Musculoskelet Disord. 2023 May 6;24:357. doi: 10.1186/s12891-023-06462-2 (PMC10163768; doi:10.1186/s12891-023-06462-2)
Supplement: Supplementary file 2 — Additional Tables. [file 12891_2023_6462_MOESM2_ESM.docx]

**Additional Table 1.** The seven-fold division of chronic pain conditions according to the International Classification of Disease (ICD-11).

| **Chronic Pain (MG30)** |
| --- |
| Chronic primary pain (MG30.0), e.g., fibromyalgia and unspecific chronic low back pain |
| Chronic cancer-related pain (MG30.1) |
| Chronic postsurgical or posttraumatic pain (MG30.2) |
| Chronic secondary musculoskeletal pain (MG30.3) |
| Chronic secondary visceral pain (MG30.4) |
| Chronic neuropathic pain (MG30.5) |
| Chronic secondary headache or orofacial pain (MG30.6) |

**Additional Table 2.** ICD-10 diagnoses (<https://icd.who.int/browse10/2019/en>) registered in SQRP deemed to be potentially compatible with a neuropathic pain condition, in falling order of frequency. Only a minority of patients were included as neuropathic (see text).

| **ICD-10 code** | **Number of patients**  **(n = 7,046)** | **Diagnoses** |
| --- | --- | --- |
| M53.1 | 2232 | Cervicobrachial syndrome |
| M54.4 | 1955 | Lumbago with sciatica |
| M54.6 | 490 | Pain in thoracic spine |
| M51.1 | 383 | Lumbar and other intervertebral disc disorders with radiculopathy |
| M51.2 | 338 | Other specified intervertebral disc displacement |
| M50.1 | 259 | Cervical disc disorder with radiculopathy |
| M79.6 | 258 | Pain in limb |
| M51.9 | 134 | Intervertebral disc disorder, unspecified |
| M48.0 | 125 | Spinal stenosis |
| M79.2 | 108 | Neuralgia and neuritis, unspecified |
| M47.8 | 98 | Other spondylosis |
| M51.3 | 83 | Other specified intervertebral disc degeneration |
| M54.1 | 68 | Radiculopathy |
| M43.1 | 67 | Spondylolisthesis |
| G62.9 | 63 | Polyneuropathy, unspecified |
| M50.9 | 57 | Cervical disc disorder, unspecified |
| G50.1 | 56 | Atypical facial pain |
| M51.1K* | 55 | Lumbar intervertebral disc disorders with radiculopathy (sciatica) |
| M796B* | 45 | Pain in upper arm |
| M47.9 | 44 | Spondylosis, unspecified |
| M51.0 | 44 | Lumbar and other intervertebral disc disorders with myelopathy |
| M47.2 | 42 | Other spondylosis with radiculopathy |
| M50.3 | 42 | Other cervical disc degeneration |

*National subclassification per the *Swedish* version of International Classification of Disease (ICD-10-SE).

**Additional Table 3**. The 22 mandatory outcome variables at baseline assessment in the two groups. Note that only a selection later participated in IPRP. * denotes significant group difference.

| Baseline | **Non-neuropathic group** |  | **Neuropathic group** |  | Statistics |  |
| --- | --- | --- | --- | --- | --- | --- |
|  | Mean | SD | Mean | SD | *P*-value | ES |
| NRS-7days | 7.10 | 1.72 | 7.17 | 1.79 | 0.099 | -0.04 |
| HADS-A | 9.41 | 4.98 | 9.12 | 5.08 | 0.027* | 0.06 |
| HADS-D | 8.73 | 4.66 | 8.90 | 4.85 | 0.165 | -0.04 |
| MPI-Pain-severity | 4.51 | 0.92 | 4.56 | 0.98 | 0.050 | -0.05 |
| MPI-Pain-interfer | 4.44 | 1.04 | 4.53 | 1.03 | 0.002* | -0.08 |
| MPI-LifeCon | 2.62 | 1.16 | 2.71 | 1.21 | 0.009* | -0.07 |
| MPI-Distress | 3.54 | 1.32 | 3.52 | 1.34 | 0.617 | 0.01 |
| MPI-Socsupp | 4.12 | 1.39 | 4.38 | 1.31 | <.001* | -0.18 |
| MPI-punish | 1.81 | 1.41 | 1.81 | 1.40 | 0.983 | 0.00 |
| MPI-protect | 3.01 | 1.45 | 3.15 | 1.43 | <.001* | -0.10 |
| MPI-distract | 2.54 | 1.23 | 2.64 | 1.24 | 0.006* | -0.08 |
| MPI-GAI | 2.38 | 0.88 | 2.25 | 0.94 | <.001* | 0.15 |
| EQ-5D-index | 0.24 | 0.31 | 0.19 | 0.31 | <.001* | 0.18 |
| EQ-VAS | 40.34 | 20.02 | 38.91 | 20.64 | 0.010* | 0.07 |
| sf36-pf | 50.50 | 21.41 | 46.79 | 21.55 | <.001* | 0.17 |
| sf36-rp | 12.79 | 24.90 | 12.26 | 25.06 | 0.435 | 0.02 |
| sf36-bp | 23.13 | 14.50 | 21.47 | 14.64 | <.001* | 0.11 |
| sf36-gh | 39.07 | 21.05 | 42.07 | 21.09 | <.001* | -0.14 |
| sf36-vt | 22.72 | 18.82 | 25.79 | 19.63 | <.001* | -0.16 |
| sf36-sf | 46.37 | 26.03 | 45.45 | 27.14 | 0.187 | 0.04 |
| sf36-re | 41.73 | 43.16 | 40.21 | 43.77 | 0.200 | 0.04 |
| sf36-mh | 54.06 | 22.39 | 53.82 | 23.33 | 0.682 | 0.01 |

ES = effect size (Hedges’ correction); NRS-7days = Pain intensity as measured by a numeric rating scale for the previous 7 days; HADS = Hospital Anxiety and Depression Scale; MPI = Multidimensional Pain Inventory; EQ-5D-index = The index of the European quality of life instrument; EQ-VAS = The European quality of life instrument thermometer-like scale; sf36 = The Short Form (36) Health Survey. See Methods for explanations of the subscale abbreviations.
